# VTP-Identifier: Vesicular Transport Proteins Identification Based on PSSM Profiles and XGBoost

**DOI:** 10.3389/fgene.2021.808856

**Published:** 2022-01-03

**Authors:** Yue Gong, Benzhi Dong, Zixiao Zhang, Yixiao Zhai, Bo Gao, Tianjiao Zhang, Jingyu Zhang

**Affiliations:** ^1^ College of Information and Computer Engineering, Northeast Forestry University, Harbin, China; ^2^ Department of Radiology, The Second Affiliated Hospital, Harbin Medical University, Harbin, China; ^3^ Department of Neurology, The Fourth Affiliated Hospital of Harbin Medical University, Harbin, China

**Keywords:** protein function prediction, vesicular transport proteins, machine learning, XGBoost, position-specific scoring matrix

## Abstract

Vesicular transport proteins are related to many human diseases, and they threaten human health when they undergo pathological changes. Protein function prediction has been one of the most in-depth topics in bioinformatics. In this work, we developed a useful tool to identify vesicular transport proteins. Our strategy is to extract transition probability composition, autocovariance transformation and other information from the position-specific scoring matrix as feature vectors. EditedNearesNeighbours (ENN) is used to address the imbalance of the data set, and the Max-Relevance-Max-Distance (MRMD) algorithm is adopted to reduce the dimension of the feature vector. We used 5-fold cross-validation and independent test sets to evaluate our model. On the test set, VTP-Identifier presented a higher performance compared with GRU. The accuracy, Matthew’s correlation coefficient (MCC) and area under the ROC curve (AUC) were 83.6%, 0.531 and 0.873, respectively.

## 1 Introduction

Researchers have paid more attention to vesicular transport proteins in recent years. Vesicular transport is that macromolecular substances or granular substances cannot pass through the cell membrane, but transport across the cell membrane in another special way, that is, substances are wrapped by the membrane, formed vesicular, fused with the membrane or broken in the process of transport in and out of the cell. Vesicular transport proteins are contained in the cell membrane, which can promote the activity of dominant molecules on the vesicle membrane. When macromolecules and particles cannot cross the cell membrane, vesicular transport proteins take on the task of transporting them. To date, many studies have confirmed that abnormal vesicular transport proteins may cause a variety of human diseases (Zhang et al., 2019; Zeng et al., 2020a), such as Hermansky-Pudlaksyndrome and chylomron retention disease (Cláudio et al., 2001; Suzuki et al., 2006). As the relationship between vesicular transport proteins and related diseases is gradually becoming clear, it is particularly important to deepen the study of vesicular transport proteins.

In view of the importance of vesicular transport proteins in eukaryotic cells, researchers in the area of cell biology have been committed to developing experimental techniques that can identify vesicular transport proteins and have achieved excellent results, such as morpholino knockdown (Hager et al., 2010) and dissection (Orci et al., 1989). These techniques can accurately identify vesicular transport proteins, but these technologies are often not very efficient and are expensive, so it is particularly necessary to find a time-saving and high accuracy method to identify vesicular transport proteins.

In recent years, protein function prediction has been a hot topic in the field of computational biology (Ding et al., 2020a; Fu et al., 2020; Guo et al., 2020; Tao et al., 2020; Wang et al., 2020; Zhai et al., 2020; Cai et al., 2021; Li et al., 2021; Yang, 2021). With the continuous enrichment of protein data, the technology of applying machine learning and data mining to protein function prediction is gradually maturing (Liu et al., 2019; Ding et al., 2020b; Ding et al., 2020c; Liu et al., 2020; Zhao et al., 2021). For example, some researchers used machine learning technology and created high accuracy models by sequence analysis (Chou, 2009; Cui et al., 2019; Jin et al., 2021; Shao et al., 2021), position-specific scoring matrix (PSSM) (Jones, 1999), and to determine various physicochemical and biochemical properties of amino acids (Kawashima and Kanehisa, 2000; Zhang et al., 2021; Zulfiqar et al., 2021). The above studies have shown that the use of computer technology in protein identification is reliable. Deep learning has attracted much attention, and researchers have been trying to create new deep neural networks to solve protein-related problems, such as the prediction of DNA-binding proteins (Qu et al., 2017), human protein subcellular localization (Wei et al., 2018a) and SNARE-CNN (Le and Nguyen, 2019). An increasing number of models and algorithms that can accurately identify proteins have been developed. Therefore, we adopted a machine learning method to obtain a model that can identify vesicular transport proteins.

In the previous study of Nguyen Quoc Khanh Le (Le et al., 2019), the strategy that includes gated recurrent units and PSSM was adopted, and the accuracy and Matthew’s correlation coefficient (MCC) of the final model reached 82.3% and 0.52 in the cross-validation set and 85.8% and 0.44 in the independent test data set, which is an excellent result. Deep learning can often achieve high accuracy, but this method will be time-consuming due to training and has a high requirement for computer equipment. Taking PSSM as input to the model for training will also increase the training time, so we hope to find a more efficient and more accurate method to identify vesicular transport proteins.

The method used in this paper extracts information such as transition probability composition, autocovariance transformation and other information from PSSM as a feature vector. We adopted undersampling, oversampling and combined sampling methods to reduce the imbalance of the data set. The Max-Relevance-Max-Distance algorithm (Zou et al., 2016) was used to sort features and reduce the number of features. In this work, we selected XGBoost as the classifier and evaluated our model with 5-fold cross-validation. Finally, we obtained a better model than a previous study, which had high efficiency and accurate identification of vesicular transport proteins.

## 2 Materials and Methods

**FIGURE 1:**
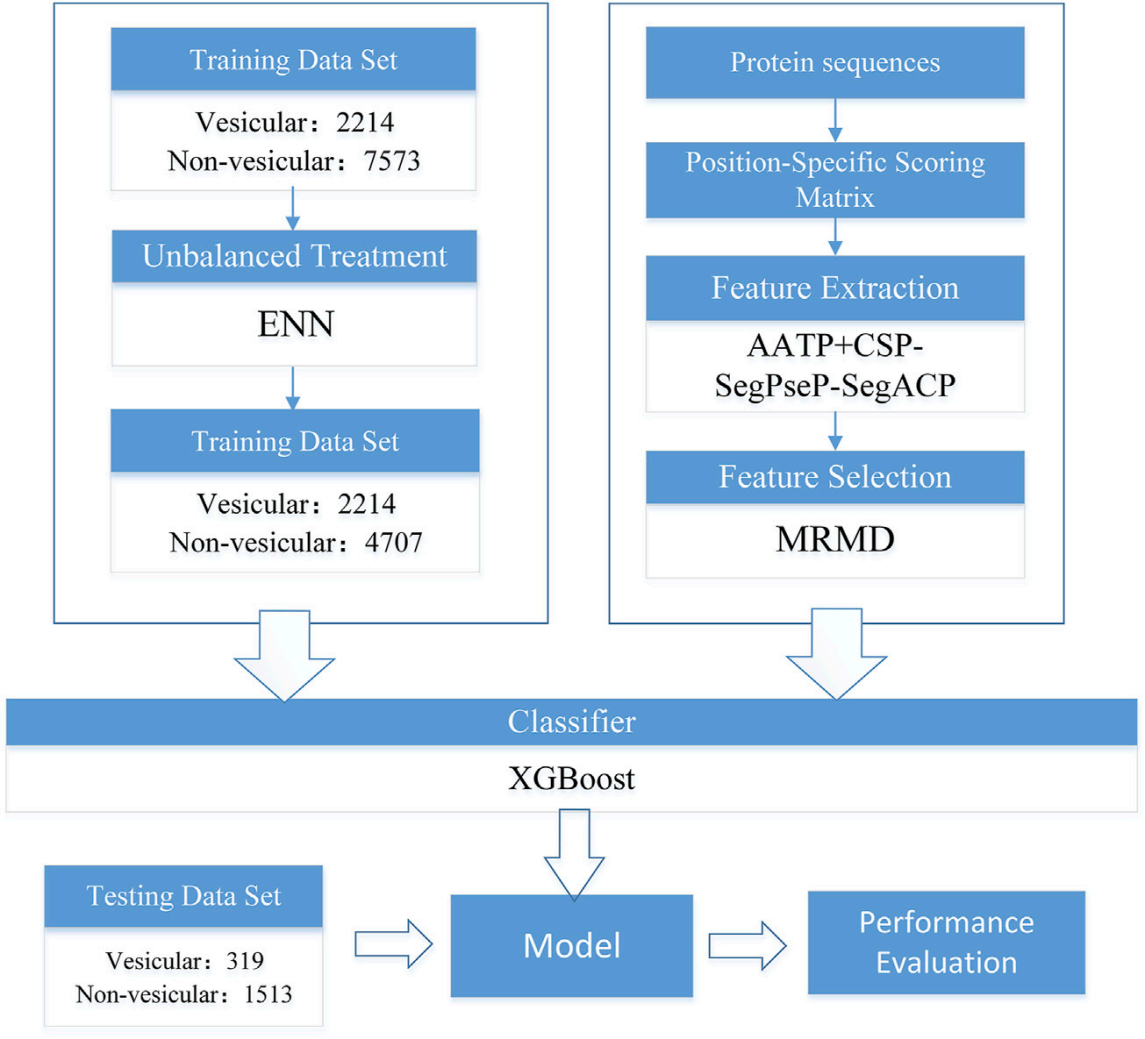
Training flow chart of the prediction model of vesicular transport proteins.

The flowchart of our work is shown in Figure 1, and each section in the figure is described in detail in the following sections.

### 2.1 Benchmark Dataset

In this work, we used the dataset provided in Nguyen Quoc Khanh Le’s study (Le et al., 2019) as the benchmark dataset. The numbers of vesicular transport proteins and non-vesicular transport proteins were 2,533 and 9,086, respectively, and we took vesicular transport proteins as positive samples and non-vesicular transport proteins as negative samples. We divided the data set into a training set and a testing set, and the details are shown in Table 1.

**TABLE 1:** Statistics of the dataset in this work.

	Total	Train	Test
Vesicular	2,533	2,214	319
Non-vesicular	9,086	7,573	1,513

### 2.2 Unbalanced Datasets Treatment

We used seven methods from an unbalanced-learning library (Lemaître et al., 2017) to address the imbalance in the dataset. The methods used for undersampling were RandomUnder, ClusterCentroids, NearMiss and EditedNearesNeighbours (ENN). The method used for over-sampling was SMOTE, a total of 5,300 positive sample data have been generated. We used default parameters for these methods. For the cleaning undersampling techniques, ENN adjusted the ratio of positive and negative samples to 1:2. The other four methods changed the number of positive and negative samples to equal. The methods used from the combined methods were SMOTEENN and SMOTETomek. We adjusted the parameters of these two methods and adjusted the proportion of positive and negative samples to 1:1.

As the SMOTE, SMOTEENN and SMOTETomek methods will generate new samples, the results of 5-fold cross-validation processed by these methods are not accurate, so special cross-validation should be performed when using these three methods. K-fold cross-validation divides the training dataset into k subdatasets; k-1 subdatasets are used to train the model, and the rest are used for validation. Our method uses SMOTE and other unbalanced data processing methods to train the k-1 subdataset and then uses the validation set to evaluate the model.

### 2.3 Feature Extraction

To date, a strategy that includes deep learning and PSSM profiles has been frequently adopted to realize the identification of unknown proteins and has achieved excellent results. However, the strategy is slightly inefficient, so in this work, we used other machine learning models and adopted RPSSM (Ding et al., 2014), CSP-SegPseP-SegACP (Liang et al., 2015), AATP (Zhang et al., 2012), DWT (Wang et al., 2017; Wang, 2019) and SOMA (Liang and Zhang, 2017) to extract features from the PSSM matrix and make a comparison. Among them, AATP and CSP-SegPseP-SegACP have the highest MCC and AUC, so they are selected as feature extraction methods.

#### 2.3.1 Position-Specific Scoring Matrix

PSSM can reveal the evolutionary information of proteins (Jones, 1999). PSSM was mainly used to predict protein secondary structure, now it has been widely used in the field of bioinformatics. Previous studies have shown that it is reliable to extract PSSM from protein sequences, and the evolutionary information in PSSM has more research value than the sequence itself (Kim and Park, 2004).

According to the definition of PSSM, we described PSSM by the following formula:

$$P_{PSSM}=\left(\begin{array}{cc} P_{1,1} & P_{1,2}\\ P_{i,1} & P_{i,2}\\ P_{L,1} & P_{L,2} \end{array}\;\;\;\;\begin{array}{cc} L & P_{1,j}\\ L & P_{i,j}\\ L & P_{L,j} \end{array}\;\;\;\begin{array}{cc} L & P_{1,20}\\ L & P_{i,20}\\ L & P_{L,20} \end{array}\right)$$

where $P_{i,j}$ represents the score of the *i*th amino acid residue of the protein sequence that mutates into amino acid type j during evolution and L shows the length of the sequence. In this work, we used PSI-BLAST to compare the sequence with NCBI’s nonredundant (NR) database to obtain PSSM. Now, many methods of extracting features from PSSM have been derived. The methods used in this paper are introduced in the following chapters.

#### 2.3.2 AATP

AATP can be extracted from PSSM, which consists of two feature vectors: amino acid composition (AAC) and transition probability composition (TPC). AAC can be described by the following:

$$AAC={\left(x_{1,}x_{1,}\ldots ,x_{20}\right)}^{T}$$

$$x_{j}=\left(\frac{1}{L}\right)\sum_{i=1}^{L}P_{i,j},j=1,2,3,\ldots ,20$$

where $x_{j}$ represents the probability that the amino acid residues change into J-type amino acids during evolution.

TPC is a feature vector of 400 dimensions that is extracted from the transition probability matrix (TPM) by:

$$TPC=\left(X_{1,1},\ldots ,X_{1,20},\ldots ,X_{i,1},\ldots X_{i,20},\ldots ,X_{20,1},\ldots ,X_{20,20}\right)$$

where

$$X=\left(\sum_{k=1}^{L-1}P_{k,i}\times P_{k+1,j}\right)/\left(\sum_{j=1}^{20}\sum_{k=1}^{L-1}P_{k+1,j}\times P_{k,i}\right),\;1\leq i,j\leq 20$$

The new feature vector AATP can be obtained by integrating AAC and TPC, and each protein sequence can extract 20 + 400 = 420 features.

#### 2.3.3 CSP-SegPseP-SegACP

CSP-SegPseP-SegACP consists of the following three parts: Pseudo-position-specific scoring matrix (PsePSSM), Autocovariance Transformation and Consensus Sequence Based on PSSM.

##### 2.3.3.1 PsePSSM

In this step, PSSM is processed twice. For the first time, PSSM was divided into two equal length segments $L_{1}$ , $L_{2}$ by using a similar procedure in (Yang and Chen, 2011). Then, two segments were used to calculate segments. The equations are as follows:

$$\alpha_{j}^{\lambda }=\{\begin{array}{l} \frac{1}{L_{1}}\sum_{i=1}^{L_{1}}P_{i,j},j=1,2,\ldots ,20,\lambda =0,\\ \frac{1}{L_{1}-\lambda }\sum_{i=1}^{L_{1}-\lambda }{\left(P_{i,j}-P_{i+\lambda ,j}\right)}^{2},j=1,2,\ldots ,20,\lambda =1,2,3,4,\;\; \end{array}$$

$$\beta_{j}^{\lambda }=\{\begin{array}{l} \frac{1}{L-L_{1}}\sum_{i=L_{1}+1}^{L}P_{i,j},j=1,2,\ldots ,20,\lambda =0,\\ \frac{1}{L-L_{1}-\lambda }\sum_{i=L_{1}+1}^{L-\lambda }{\left(P_{i,j}-P_{i+\lambda ,j}\right)}^{2},j=1,2,\ldots ,20,\lambda =1,2,3,4,\;\;\;\; \end{array}$$

where $\alpha_{j}^{\lambda }$ and $\beta_{j}^{\lambda }$ represent the correlation between amino acids and λ is the contiguous distance of $\alpha_{j}^{\lambda }$ and $\beta_{j}^{\lambda }\;$ along the protein sequence of each fragment. The value range of λ is affected by the number of PSSM segments and the length of the shortest series, so λ can be taken to be 0, 1, 2, 3 and 4. Through the above calculation, we can obtain a 200-dimensional feature vector.

Next, the PSSM is divided into three segments $L_{1}$ , $L_{2}$ and $L_{3}$ ; here, λ can be token to 0, 1 and 2. The equations are as follows:

$$\theta_{j}^{\lambda }=\{\begin{array}{l} \frac{1}{L_{1}}\overset{L_{1}}{\underset{i=1}{\sum }}P_{i,j},j=1,2,\ldots ,20,\lambda =0,\;\;\\ \frac{1}{L_{1}-\lambda }\overset{L_{1}-\lambda }{\underset{i=1}{\sum }}\sum_{i=1}^{L_{1}-\lambda }{\left(P_{i,j}-P_{i+\lambda ,j}\right)}^{2},j=1,2,\ldots ,20,\lambda =1,2\; \end{array}$$

$$\mu_{j}^{\lambda }=\{\begin{array}{l} \frac{1}{L_{1}}\sum_{i=L_{1}+1}^{2L_{1}}P_{i,j},j=1,2,\ldots ,20,\lambda =0,\\ \frac{1}{L_{1}-\lambda }\sum_{i=L_{1}+1}^{2L_{1}-\lambda }{\left(P_{i,j}-P_{i+\lambda ,j}\right)}^{2},j=1,2,\ldots ,20,\lambda =1,2 \end{array}$$

$$\nu_{j}^{\lambda }=\{\begin{array}{l} \frac{1}{L-2L_{1}}\sum_{i=L_{1}+1}^{L_{1}}P_{i,j},j=1,2,\ldots ,20,\lambda =0,\\ \frac{1}{L-2L_{1}-\lambda }\sum_{i=2L_{1}+1}^{L-\lambda }{\left(P_{i,j}-P_{i+\lambda ,j}\right)}^{2},j=1,2,\ldots ,20,\lambda =1,2 \end{array}\;$$

This time, 180-dimensional feature vectors are obtained. Combined with the results of the previous stage, a 380-dimensional feature vector can be extracted from PSSM.

##### 2.3.3.2 Autocovariance Transformation

In this step, the information contained in the sequence is further extracted by calculating the autocovariance transformation. Similar to the previous step, the PSSM is divided into two segments and three segments, and then the ACT-PSSM feature vector is obtained by the following equations when divided into two segments:

$$AC1_{j}^{\mathrm{lg}}=\frac{1}{L_{1}-l_{g}}\sum_{i=1}^{L_{1}-l_{g}}\left(P_{i,j}-\alpha_{j}^{0}\right)\left(P_{i+l_{g},j}-\alpha_{j}^{0}\right),j=1,2,\ldots ,20,l_{g}=1,2,3,4$$

$$AC2_{j}^{\mathrm{lg}}=\frac{1}{L-L_{1}-l_{g}}\sum_{i=L_{1}+1}^{L-l_{g}}\left(P_{i,j}-\beta_{j}^{0}\right)\left(P_{i+l_{g},j}-\beta_{j}^{0}\right),j=1,2,\ldots ,20,l_{g}=1,2,3,4$$

$$AC1_{j}^{\mathrm{lg}}=\frac{1}{L_{1}-l_{g}}\sum_{i=1}^{L_{1}-l_{g}}\left(P_{i,j}-\theta_{j}^{0}\right)\left(P_{i+l_{g},j}-\theta_{j}^{0}\right),j=1,2,\ldots ,20,l_{g}=1,2$$

$$AC2_{j}^{\mathrm{lg}}=\frac{1}{L_{1}-l_{g}}\sum_{i=L_{1}+1}^{2L_{1}-l_{g}}\left(P_{i,j}-\mu_{j}^{0}\right)\left(P_{i+l_{g},j}-\mu_{j}^{0}\right),j=1,2,\ldots ,20,l_{g}=1,2$$

$$AC3_{j}^{\mathrm{lg}}=\frac{1}{L-2L_{1}-l_{g}}\sum_{i=2L_{1}+1}^{L-l_{g}}\left(P_{i,j}-\nu_{j}^{0}\right)\left(P_{i+l_{g},j}-\nu_{j}^{0}\right),j=1,2,\ldots ,20,l_{g}=1,2$$

where $l_{g}$ represents the differences between amino acid residues. Finally, the 280-dimensional ACT-PSSM feature vector can be obtained by the above equations.

##### 2.3.3.3 Consensus Sequence Based on PSSM

This step adopts the method in (Patthy, 1987) and generates a consensus sequence as follows:

$$X\left(i\right)=\arg\max\left\{P_{i,j}:1\leq j\leq 20\right\},\;1\leq i\leq L$$

Next, we compute CSAAC, which shows 20 amino acid composition features of the consensus sequence, and CSCM, which represents 20 composition moment features for CS. Through the combination of the above two feature vectors, we obtain a 40-dimensional feature vector based on CS.

The 700-dimensional CSP-SegPseP-SegACP feature vector is obtained by fusing the features obtained from the above three steps.

### 2.4 Feature Selection

In this section, we adopted Max-Relevance-Max-Distance algorithm (MRMD) (Zou et al., 2016) to reduce the dimension of the feature vector, MRMD uses the Pearson correlation coefficient to balance the correlation between the subfeature set and the target class and uses various distance functions to obtain the redundancy of each subfeature set. The subfeature set selected by MRMD has low redundancy and strong correlation with the target class.

### 2.5 Classification

We compared the performance of four different popular classification methods which are the RF, SVM, KNN and XGBoost to identified VTP. Due to six performance evaluations on the training set, we chose XGBoost as our classification method.

XGBoost (Chen and Guestrin, 2016) is a machine learning method with an excellent classification effect and high efficiency that has been widely used in recent years(Long et al., 2021; Yang et al., 2021). It stands out from many of the challenges of machine learning and data mining. In this paper, XGBoost performed very well, and it still obtained good results under the premise of high training efficiency.

## 3 Results

### 3.1 Assessment of Predictive Ability

In this work, our goal was to obtain a model to predict whether the unknown type of protein sequence belongs to vesicular transport proteins, so we took vesicular transport proteins in the data set as positive samples and non-vehicular transport proteins as negative samples. In each section of our work, to evaluate our model, we used 5-fold cross-validation several times and calculated the average value as the final result. After obtaining the results of cross-validation, we used a test data set to test our model and make adjustments.

To evaluate our model comprehensively, we used several methods, including accuracy (ACC), sensitivity (Sens), specificity (Spec), precision, Matthew’s correlation coefficient (MCC) and area under the ROC curve (AUC) (Jiang et al., 2013; Wei et al., 2014; Wei et al., 2017; Wei et al., 2018b; Su et al., 2019; Zeng et al., 2020b; Hong et al., 2020; Su et al., 2020; Tang et al., 2020; Dao, 2021; Shao and Liu, 2021; Wang, 2021). These methods are defined as follows:

$$ACC=\frac{TP+TN}{TP+FN+FP+TN}$$

$$Sens=\frac{TP}{TP+FN}$$

$$Spec=\frac{TN}{FP+TN}$$

$$\mathrm{Pr}ecision\;=\frac{TP}{TP+FP}$$

$$MCC=\frac{TP\times TN-FP\times FN}{\sqrt{\left(TP+FP\right)\left(TP+FN\right)\left(TN+FP\right)\left(TN+FN\right)}}$$

$$AUC=\frac{1}{2}\left(\frac{TP}{TP+FN}+\frac{TN}{TN+FP}\right)$$

where TP, FP, TN and FN represent true positives, false positives, true negatives, and false negatives, respectively.

### 3.2 Comparison of the Different Unbalanced Data Processing Methods

In the previous section, we selected CSP-SegPseP-SegACP and AATP as the feature extraction methods. Next, we compared the effects of different unbalanced processing methods on the model.

When the samples are in an unbalanced state, the model trained by machine learning tends to be more inclined to a large number of samples. (Fdez-Glez et al., 2018).

We used seven methods from an unbalanced-learning library to address the imbalance in the data set. The methods are RandomUnder, ClusterCentroids, NearMiss, EditedNearesNeighbours (ENN), SMOTE, SMOTEENN and SMOTETomek. The RandomUnder, ClusterCentroids and NearMiss adjusted the number of positive and negative samples to 2214:2214. The ENN adjusted the number of positive and negative samples to 2214:4707. The SMOTE adjusted the number of positive and negative samples to 7573:7573. The SMOTEENN and SMOTETomek adjusted the number of positive and negative samples to 5000:5000.

**FIGURE 2:**
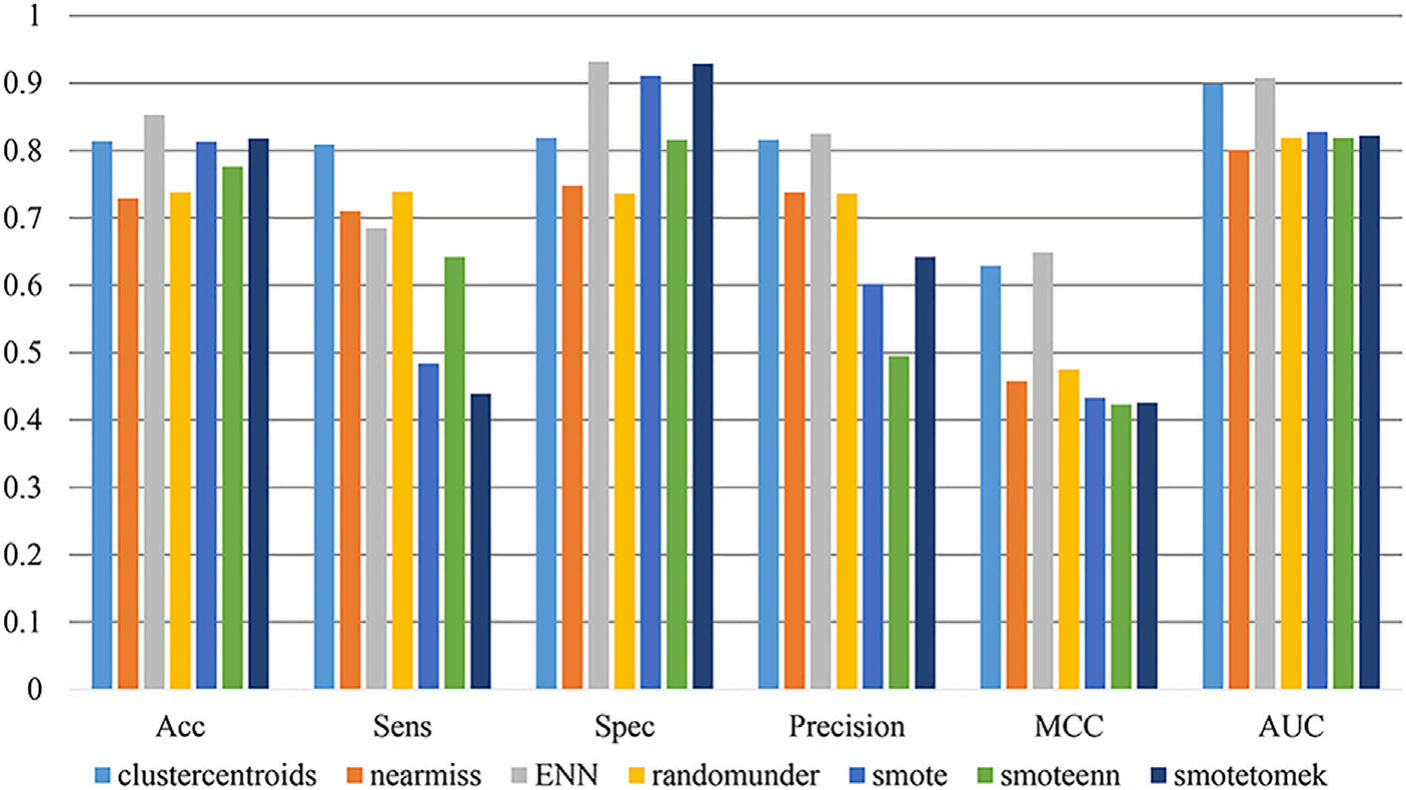
The values of the different unbalanced data processing methods on the training set.

In this part of our work, we set the XGBoost parameter scale_pos_weight = default to avoid XGBoost training being more biased towards positive samples. Other parameters of XGBoost are set as follows: learning_rate = 0.1, n_estimators = 1,000, max_depth = 8, min_child_weight = 1, gamma = 0, subsample = 0.8, colsample_bytree = 0.8, objective = “binary:logistic”, nthread = 20. We found that the ENN method is the best, and its ACC, MCC, AUC and so on are significantly higher than those of the other methods. Therefore, ENN was selected as the final unbalanced data processing method. The result on the training set after using different imbalance processing algorithms is shown in Figure 2.

When dealing with unbalanced data, we cannot precisely control the proportion of positive and negative samples when using ENN. The dataset was still slightly unbalanced, so we continued to adjust the parameter scale_pos_weight of XGBoost, which makes the classifier tend to have small samples in the training process. Finally, we set scale_pos_weight = 0.6. The performance of the model is shown in Table 2.

**TABLE 2:** Evaluation of model performance after processing unbalanced data by ENN.

	Acc	Sens	Spec	Precision	MCC	AUC
ENN	0.85	0.701	0.919	0.811	0.659	0.908

### 3.3 Comparison of the Different Feature Extraction Methods

In previous studies, the training model using PSSM as input can effectively predict vesicular transport proteins, which indicates that PSSM has important information to identify vesicular transport proteins. In this paper, the methods of extracting features from PSSM were used to further extract the key information in PSSM and to improve the efficiency of the training model.

**FIGURE 3:**
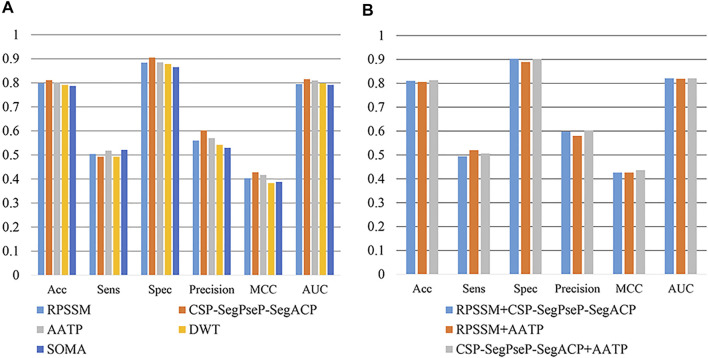
**(A)** Comparison of single feature extraction methods. **(B)** Comparison of combining feature extraction methods.

In this section, RPSSM, AATP, CSP-SegPseP-SegACP, SOMA, and DWT were used to extract features from PSSM. In addition to different feature extraction methods, other experimental conditions are completely consistent. We adopted XGBoost as the classifier, set the scale_pos_weight = 0.1 for the temporary method for dealing with unbalanced data sets, and used cross validation to evaluate our model. The result is shown in Figure 3A. By comparison, we found that RPSSM, CSP-SegPseP-SegACP and AATP performed better.

Next, we combined these three methods in pairs for comparison. We found that the combination of CSP-SegPseP-SegACP and AATP was the best method, through these two methods, we extracted 1,120 dimension feature vectors. The result after using the combination methods on the training set is shown in Figure 3B.

### 3.4 Feature Selection

After dealing with the imbalance of the data set, our model has made significant progress. In this section, we reduced the dimension of the feature vector by feature selection.

In the process of machine learning, the high dimensionality of the input feature vector will have a huge impact on the model, which will make the model too complex and reduce the generalization. Therefore, when the dimension of the feature vector is high, dimensionality reduction can improve the learning ability of the machine learning model and reduce the time required to train the model.

In this work, we adopted Max-Relevance-Max-Distance algorithm (MRMD). By using AATP and CSP-SegPseP-SegACP to extract features, and then combined the features and normalized them by Z-score standardization. The dimension of the feature vector is 1,120. In this work, we used the latest version of MRMD to improve our model. MRMD has five feature ranking methods: Hits_a, Hits_h, TrustRank, PageRank and LeaderRank. TrustRank and PageRank were originally used in web search system, MRMD modified them and applied them to feature selection. LeaderRank is derived from the basic PageRank algorithm. It adds a background node to make two-way connection with all nodes. Hits is similar to PageRank and is also applied to web search, the difference is that the number of web pages processed by hits is small, and it is related to queries. We used all five methods, and then we chose Hits_h by comparing the results of cross-validation. The results are shown in Table 3. Finally, through MRMD, we changed the 1120-dimensional feature vector to 791 dimensions, and the accuracy was also improved.

**TABLE 3:** The results of using different sorting methods in MRMD on the training set.

	Dimension	Acc	Sens	Spec	Precision	MCC	AUC
Hits_a	681	0.852	0.711	0.919	0.805	0.653	0.907
TrustRank	992	0.857	0.709	0.927	0.818	0.658	0.907
PageRank	898	0.855	0.712	0.922	0.81	0.658	0.907
LeadeRank	738	0.854	0.712	0.921	0.809	0.656	0.908
Hits_h	791	0.855	0.713	0.921	0.813	0.66	0.908

### 3.5 Performance on Different Methods

Through the above processing, we obtained a good performance model. In this model, the parameters of XGBoost are: learning_rate = 0.1, n_estimators = 1,000, max_depth = 8, min_child_weight = 1, gamma = 0, subsample = 0.8, colsample_bytree = 0.8, objective = “binary:logistic”, nthread = 20, scale_pos_weight = 0.6. Next, we compared the effect of our cross-validation set on different methods. In this section, we applied the data processed by the same feature extraction method, imbalance processing method and feature selection method to different machine learning models.

**FIGURE 4:**
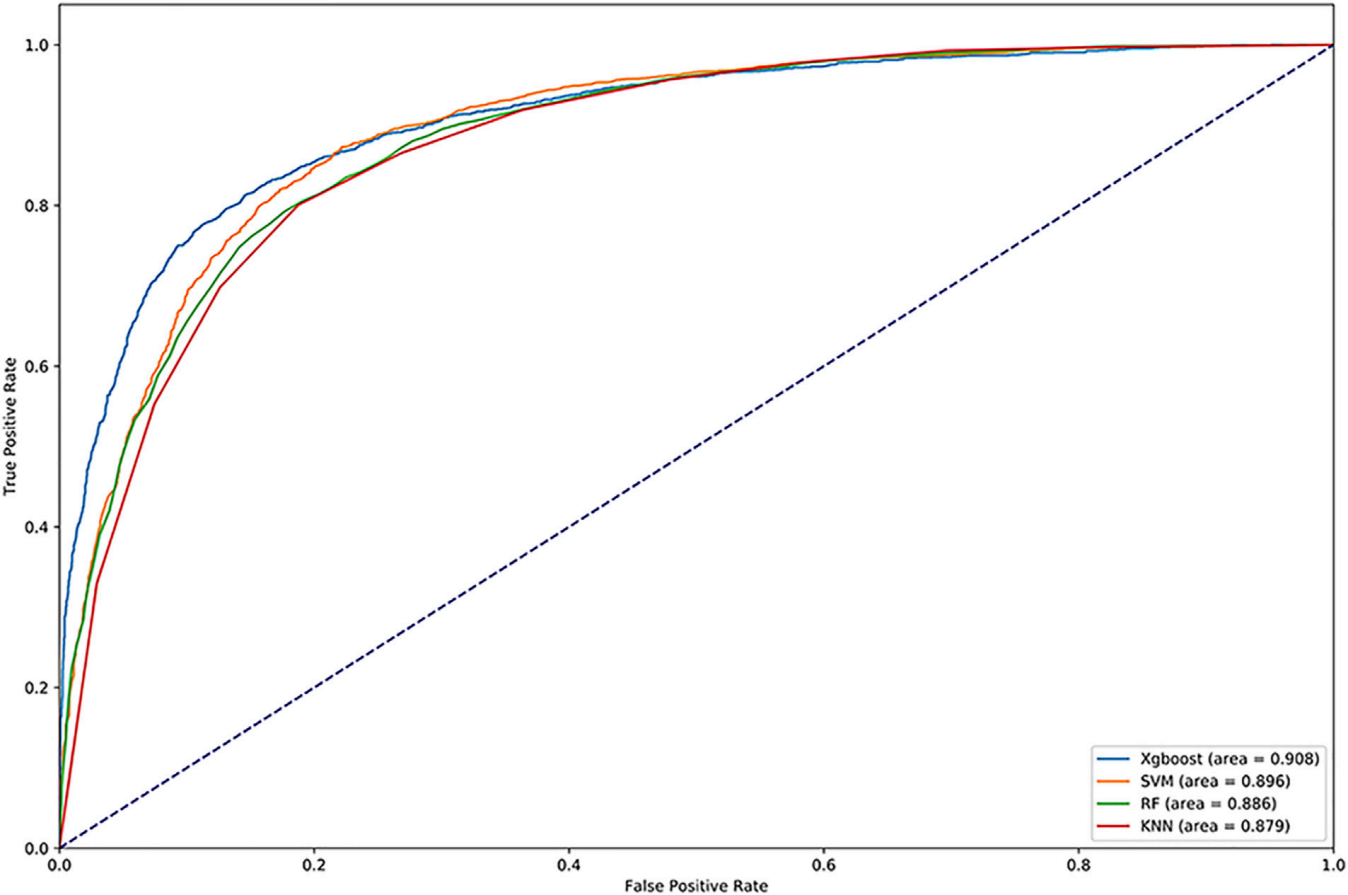
ROC curve of vesicle transporters identified by different methods.

We used Random Forest, KNN and SVM for comparison. We optimized the parameters of each classifier and set n_estimators = 100 in random forest, k = 10 in KNN, gamma = 0.5 and cost = 8 in SVM. The results are shown in Table 4. We drew the ROC and calculated the AUC, which are shown in Figure 4. Obviously, XGBoost is the best choice. Compared with other methods, XGBoost was also very efficient in the process of training the model.

**TABLE 4:** Comparison of six performance evaluations on the training set.

	Acc	Sens	Spec	Precision	MCC	AUC
RF	0.823	0.582	0.936	0.81	0.574	0.886
SVM	0.843	0.72	0.9	0.773	0.633	0.896
KNN	0.822	0.732	0.865	0.72	0.595	0.879
XGBoost	0.855	0.713	0.921	0.813	0.66	0.908

**FIGURE 5:**
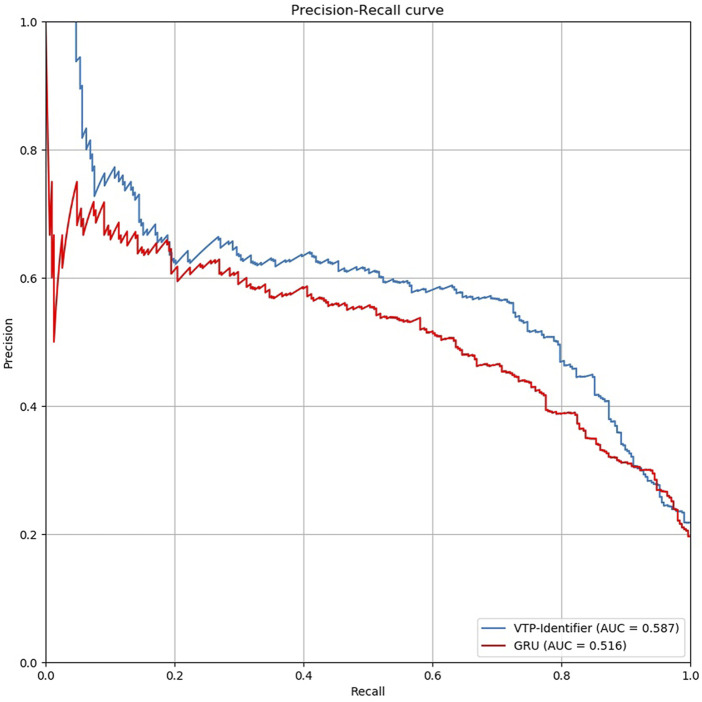
Comparison of PR curves between our model and GRU.

Then, we used independent test sets to test the model performance. Nguyen Quoc Khanh Le used Gru neural network for deep learning in his research, we used the model provided by Nguyen Quoc Khanh Le’s research result to classify vesicular transport proteins, and then compared with our model. The results are shown in Table 5. The PR curves of the two models are shown in Figure 5. Obviously, the performance of our model is better.

**TABLE 5:** Performance comparison between our model and GRU.

	Acc	Sens	Spec	Precision	MCC	AUC
GRU	0.809	0.708	0.829	0.515	0.459	0.850
VTP-Identifier	0.836	0.757	0.852	0.517	0.531	0.873

## 4 Discussion

In this paper, we provide a method to identify vesicular transport proteins based on feature extraction from PSSM. In our dataset, the number of vesicular transport proteins and non-vesicular transport proteins are 2,533 and 9,086, and the number of training sets are 2,214 and 7,573. We used ENN to address the imbalance of the training data set, reduced the number of non-vesicular transport proteins from 7,573 to 4,707. We used AATP and CSP-SegPseP-SegACP to extract features from PSSM and then obtained 1,120 dimensional feature vector. Next we used MRMD to reduce the dimension of the feature vector and the dimension is reduced to 791. Finally, we sent the processed data to XGBoost and got a model to accurately identify vesicular transport proteins. The experimental comparison shows that our model is better than the previous research result. The accuracy of our model on the test set is 83.6%, which exceeds the previous research results obtained by Nguyen Quoc Khanh Le through deep learning.

## Data Availability

The datasets presented in this study can be found in online repositories. The names of the repository/repositories and accession number(s) can be found below: https://github.com/1024488648/vesicle_transporter.
